# Machine learning to differentiate small round cell malignant tumors and non-small round cell malignant tumors of the nasal and paranasal sinuses using apparent diffusion coefficient values

**DOI:** 10.1007/s00330-021-08465-w

**Published:** 2022-01-14

**Authors:** Chen Chen, Yuhui Qin, Haotian Chen, Junying Cheng, Bo He, Yixuan Wan, Dongyong Zhu, Fabao Gao, Xiaoyue Zhou

**Affiliations:** 1 Molecular Imaging Laboratory, Department of Radiology, West China Hospital, Sichuan University, 37 Guoxue Road, Chengdu, Sichuan 610041 People’s Republic of China; 2 Department of MRI, the First Affiliated Hospital of Zhengzhou University, Zhengzhou, People’s Republic of China; 3 MR Collaboration, Siemens Healthineers Ltd., Shanghai, People’s Republic of China

**Keywords:** Apparent diffusion coefficient, Radiomics, Machine learning, Neoplasms

## Abstract

**Objective:**

We used radiomics feature–based machine learning classifiers of apparent diffusion coefficient (ADC) maps to differentiate small round cell malignant tumors (SRCMTs) and non-SRCMTs of the nasal and paranasal sinuses.

**Materials:**

A total of 267 features were extracted from each region of interest (ROI). Datasets were randomized into two sets, a training set (∼70%) and a test set (∼30%). We performed dimensional reductions using the Pearson correlation coefficient and feature selection analyses (analysis of variance [ANOVA], relief, recursive feature elimination [RFE]) and classifications using 10 machine learning classifiers. Results were evaluated with a leave-one-out cross-validation analysis.

**Results:**

We compared the AUC for all the pipelines in the validation dataset using FeAture Explorer (FAE) software. The pipeline using RFE feature selection and Gaussian process classifier yielded the highest AUCs with ten features. When the “one-standard error” rule was used, FAE produced a simpler model with eight features, including Perc.01%, Perc.10%, Perc.90%, Perc.99%, S(1,0) SumAverg, S(5,5) AngScMom, S(5,5) Correlat, and WavEnLH_s-2. The AUCs of the training, validation, and test datasets achieved 0.995, 0.902, and 0.710, respectively. For ANOVA, the pipeline with the auto-encoder classifier yielded the highest AUC using only one feature, Perc.10% (training/validation/test datasets: 0.886/0.895/0.809, respectively). For the relief, the AUCs of the training, validation, and test datasets that used the LRLasso classifier using five features (Perc.01%, Perc.10%, S(4,4) Correlat, S(5,0) SumAverg, S(5,0) Contrast) were 0.892, 0.886, and 0.787, respectively. Compared with the RFE and relief, the results of all algorithms of ANOVA feature selection were more stable with the AUC values higher than 0.800.

**Conclusions:**

We demonstrated the feasibility of combining artificial intelligence with the radiomics from ADC values in the differential diagnosis of SRCMTs and non-SRCMTs and the potential of this non-invasive approach for clinical applications.

**Key Points:**

*• The parameter with the best diagnostic performance in differentiating SRCMTs from non-SRCMTs was the Perc.10% ADC value.*

*• Results of all the algorithms of ANOVA feature selection were more stable and the AUCs were higher than 0.800, as compared with RFE and relief.*

*• The pipeline using RFE feature selection and Gaussian process classifier yielded the highest AUC.*

## Introduction

Malignant tumors of the nasal and paranasal sinuses are rare, comprising less than 1% of all malignancies and about 3% of head and neck malignancies [1, 2]. This tumor group includes small round cell malignant tumors (SRCMTs) and non-SRCMTs. SRCMTs constitute a specific group of malignancies in the nasal and paranasal sinuses based on neuroectodermal differentiation, soft tissue differentiation, and hematopoietic differentiation. Rhabdomyosarcoma (RMS), malignant melanoma (MM), olfactory neuroblastoma (ONB), neuroendocrine carcinoma (NEC), and lymphoma are included in this group. Non-SRCMTs constitute another common group of malignant tumors in the nasal and paranasal sinuses based on epithelial differentiation and include squamous cell carcinoma (SCC) and adenoid cystic carcinoma (ACC) [3]. Distinguishing these two groups is elemental because some are managed primarily with radiation, whereas others are managed solely with chemotherapy. Still others are managed with conservative medical therapy, local surgery, exenterative surgery, and multimodal therapy, indicating that therapeutic decisions, surgical planning, and prognoses are different for the various tumor types and management strategies [4].

Varying according to the pathology and cellularity of the tissue because of the limited diffusion of water molecules, apparent diffusion coefficient (ADC) values have been used to discriminate malignant from benign nasal and paranasal sinus tumors and to differentiate various histopathologic types of malignant sinonasal tumors [5–10]. However, conventional magnetic resonance imaging (MRI) has limitations of its own when differentiating between SRCMTs and non-SRCMTs. Under the circumstances, as texture analysis (TA) techniques, by using mathematically defined features[11], can analyze pixel distributions, intensities, and dependencies, it can provide a wealth of information beyond what can be seen with the human eye and thus can be used to characterize SRCMTs and non-SRCMTs, quantitatively.

As a branch of artificial intelligence, machine learning (ML) includes various algorithms that can enhance diagnosis, treatments, and follow-up results in neuro-oncology medicine by analyzing huge complex datasets [12, 13]. More importantly, not depending on user experience, ML is more objective than other conventional analyses and has good repeatability. To achieve the optimal predictive ability and clinical utility, in the present study, we compared three feature selection methods and an array of ML algorithms. To our knowledge, no studies using TA and ML for differentiating sinonasal SRCMTs from non-SRCMTs have been reported. To bridge this gap, this retrospective study was intended to evaluate the potential value of the ML-based ADC texture analysis for distinguishing SRCMTs from non-SRCMTs by using various state-of-the-art ML algorithms.

## Materials and methods

### Patients

We used the surgical pathology database from January 1, 2018, to November 1, 2020, at our hospital. Exclusion criteria were (1) patients who received treatments before surgery and (2) inadequate image quality. All the methods were performed in accordance with the relevant guidelines and regulations, and informed consent was waived. This study was approved by the Institutional Ethics Review Committee of our hospital.

### Image acquisition

Patients were examined with a 3-T MR scanner (Siemens Skyra) with standard head coil. MRI scan protocols included the following: axial T2WI (TR/TE= 5000/117 ms, matrix=256 x 256, field of view=24 x 24cm, thickness=5 mm and intersection gap =1mm); axial DWI (spin echo-echo planar imaging) (b = 0 and 1000 s/mm^2^, TR/TE = 3200/70 ms, matrix = 160 × 160, flip angle 90°, field of view = 24 × 24 cm, thickness = 5 mm, intersection gap = 1 mm).

### Textural feature extractions

**Fig. 1:**
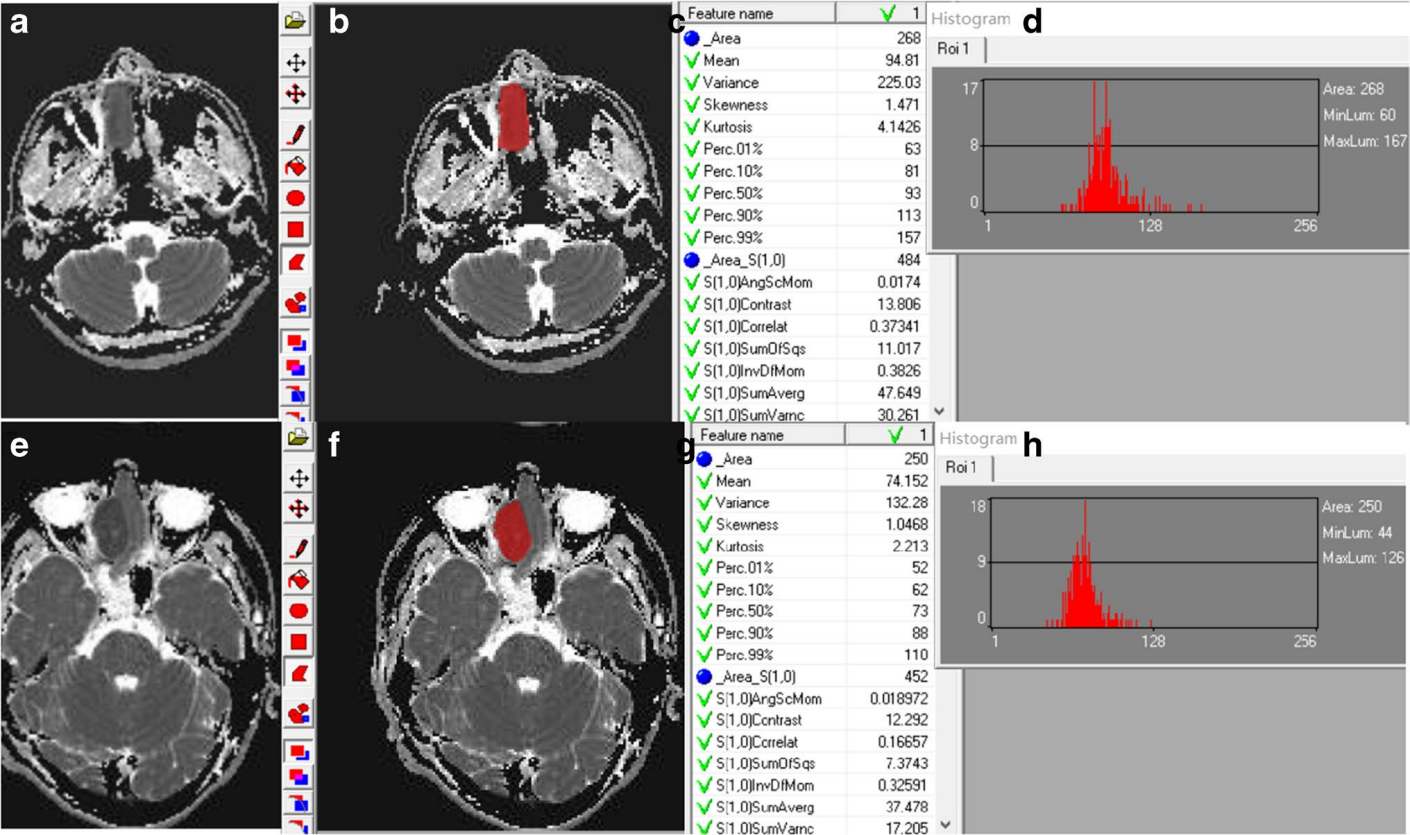
**a** shows axial ADC of a 36-year-old male patient with SCC. **b** Corresponding ROI, (**c)** parts of 267 feature values, and (**d)** histogram maps are shown. (**e)** shows axial ADC of a 44-year-old male patient with ONB. (**f)** Corresponding ROI, (**g)** some of 267 feature values, and (**h)** histogram maps are shown

MaZda v. 4.7 software (The Technical University of Lodz, Institute of Electronics, http://www.eletel.p.lodz.pl/mazda/) was used for the analyses. We used the limitation of dynamics to *μ* ± 3*δ* (*μ*: mean gray-level value, *δ*: standard deviation) [14] to achieve reliable results regarding MRI texture classifications. Regions of interest (ROIs) were drawn on ADC images. The largest lay was selected using a T2WI image reference. Two physicians with more than 10 years of experience delineated the ROIs manually along the lesion edges, and the lesion was filled in with a red marker, excluding various necrotic and cystic regions (Fig. 1). In total, 267 feature values and corresponding histogram maps were extracted for each ROI. The number of radiomics features based on feature classes is presented in Table 1, including (i) nine histogram features based on the pixel counts in an image with a specific gray-level value [15], (ii) 220 Gy-level co-occurrence matrix (GLCM) features based on the extracted statistical information about the distribution of pixel pairs [16], (iii) 20 Gy-level run-length matrix (GLRLM) features obtained by searching the image for runs having the same gray-level value in a pre-defined direction [17], (iv) 5 auto-regressive model (ARM) features based on the weights associated with four neighboring pixels and the variance of the minimized prediction error, (v) 8 wavelet transform (WAV) features on texture frequency components extracted from the energies computed within the channels [18], and (vi) 5 absolute gradient statistics (AGS) features based on the spatial variation of gray-level values across the image [15]. Multiple GLCMs were computed into the 0°, 45°, 90°, 135°, and *z*-axis directions and 1, 2, 3, and 4 pixels. Multiple GLRLMs were computed along four different angles (horizontal, vertical, diagonal 45, and diagonal 135).

**Table 1:** Texture analysis methods and the corresponding texture features

Method	Texture feature parameters
Histogram (9)	Mean, variance, skewness, kurtosis, and percentiles (1%, 10%, 50%, 90%, and 99%)
Gray-level co-occurrence matrix (GLCM) (220)	Angular second moment (AngScMom), contrast, inverse different moment (IDM), entropy (Ent), correlation (Correlat), sum of squares (SumOfSqs), sum average (SumAverg), sum variance (SumVarnc), sum entropy (SumEntrp), difference variance (DifVarnc), difference entropy (DifEntrp) along the 0°, 45°, 90°, 135°, and *z*‐axis directions and 1, 2, 3, and 4 pixels
Gray‐level run‐length matrix (GLRLM) (20)	Run-length nonuniformity (RLNonUni), gray-level nonuniformity (GLevNonU), long run emphasis (LngREmph), short run emphasis (ShrtREmp), fraction of image in runs (Fraction) of four different angels (horizontal, vertical, diagonal 45, and digonal135)
Auto‐regressive model (ARM) (5)	Teta1, Teta2, Teta3, Teta4, Sigma
Wavelet transform (WAV) (8)	Energy computed from the low–low frequency band within the first image scale (WavEnLL_s-1), WavEnLH_s-1, WavEnHL_s-1, WavEnHH_s-1, WavEnLL_s-2, WavEnLH_s-2, WavEnHL_s-2, WavEnHH_s-2
Absolute gradient statistics (AGS) (5)	Absolute gradient mean (GrMean), variance (GrVariance), skewness (GrSkewness), kurtosis (GrKurtosis), nonzeros (GrNonZeros)

### Feature selections

Computer-generated random datasets were used to assign 70% of the datasets to the training set and the rest (30%) of the datasets to the independent test set. FeAture Explorer software (FAE; V 0.3.6) was developed using the Python programming language (3.7.6) (https://github.com/salan668/FAE). First, the synthetic minority oversampling technique (SMOTE) was used to balance the training dataset. This method worked by taking each minority class sample, introducing synthetic examples along the line segments and joining any or all of the nearest *k* minority class neighbors. The neighboring points were randomly chosen depending on the amount of oversampling required. The dataset was normalized using *Z*-score normalization, which subtracted the mean value and divided the standard deviation for each feature. Second, a Pearson correlation coefficient (PCC) was used for each pair of features to reduce the dimensions of the row space of the feature matrix [19]. If the PCC was above 0.99, one of features was randomly removed. Lastly, the analysis of variance (ANOVA), relief [20], and recursive feature elimination (RFE) were utilized for feature selections. ANOVA is a common method that explores the significant features corresponding to the labels. Relief selects sub-datasets and finds relative features according to the recursive labels. RFE is intended to select features based on a classifier by recursively considering a smaller set of features. The range of the number of features was set from 1 to 20.

### Classifications

The classification performances were tested with 10 ML algorithms based on Python code with scikit-learn library (https://scikit-learn.org/), including the support vector machine (SVM), linear discriminant analysis (LDA), auto-encoder (AE), random forests (RF), logistic regression (LR), logistic regression via Lasso (LRLasso), ada-boost (AB), decision tree (DT), Gaussian process (GP), and naive Bayes (NB) (Table 2). SVM searches for an optimal separating hyperplane between classes, which maximizes the margin. C stands for regularization parameter. The strength of the regularization is inversely proportional to C. AE classification is based on neural networks (NN), which is a network of highly interconnected processing units that process information by their dynamic state responses to external inputs. LDA with a linear decision boundary was generated by fitting class conditional densities to the data and using Bayes’ rule. Solver uses singular value decomposition recommended for data with a large number of features. RF consists of a large number of individual decision trees that operate as an ensemble. Each individual tree outputs a class prediction and the class with the most votes represents the model’s prediction. The number of trees in the forest was 100. In most cases, the larger the number, the better the performance. LR explains the relationship between one dependent binary variable and one or more independent variables regressing for the probability of a categorical outcome using a logistic function. Lasso-LR is able to get a better model which can do high-dimensional statistics. Alpha is equivalent to an ordinary least square with defaults to 1.0. AB generates H hypotheses through an ensemble of learning algorithms. The output of the learning algorithms is incorporated into a weighted sum that represents the final output of the boosted classifier. DTs of supported criteria are “gini” for the Gini impurity and “entropy” for the information gain. GP was based upon Laplace approximation. The kernel was none, specifying the covariance function of the GP. NB applies Bayes’ theorem with the naive assumption of conditional independence between the features. Setting alpha = 1.0 is called Laplace smoothing.

**Table 2:** The parameters of the algorithms

Algorithms	Parameters
SVM	C = 1.0, kernel = ‘rbf’, degree = 3, gamma = ‘scale’, coef0 = 0.0, shrinking = True, probability = False, tol = 0.001, cache_size = 200, class_weight = None, verbose = False, max_iter =—1, decision_function_shape = ‘ovr’, break_ties = False, random_state = None
AE	hidden_layer_sizes = (100), activation = ‘relu’, *, solver = ‘adam’, alpha = 0.0001, batch_size = ‘auto’, learning_rate = ‘constant’, learning_rate_init = 0.001, power_t = 0.5, max_iter = 200, shuffle = True, random_state = None, tol = 0.0001, verbose = False, warm_start = False, momentum = 0.9, nesterovs_momentum = True, early_stopping = False, validation_fraction = 0.1, beta_1 = 0.9, beta_2 = 0.999, epsilon = 1e-08, n_iter_no_change = 10, max_fun = 15,000
LDA	solver = ‘svd’, shrinkage = None, priors = None, n_components = None, store_covariance = False, tol = 0.0001
RF	n_estimators = 100, *, criterion = ‘gini’, max_depth = None, min_samples_split = 2, min_samples_leaf = 1, min_weight_fraction_leaf = 0.0, max_features = ‘auto’, max_leaf_nodes = None, min_impurity_decrease = 0.0, min_impurity_split = None, bootstrap = True, oob_score = False, n_jobs = None, random_state = None, verbose = 0, warm_start = False, class_weight = None, ccp_alpha = 0.0, max_samples = None
LR	penalty = ‘l2’, *, dual = False, tol = 0.0001, C = 1.0, fit_intercept = True, intercept_scaling = 1, class_weight = None, random_state = None, solver = ‘lbfgs’, max_iter = 100, multi_class = ‘auto’, verbose = 0, warm_start = False, n_jobs = None, l1_ratio = None
LRLasso	alpha = 1.0, *, fit_intercept = True, normalize = False, precompute = False, copy_X = True, max_iter = 1000, tol = 0.0001, warm_start = False, positive = False, random_state = None, selection = ‘cyclic’
AB	base_estimator = None, *, n_estimators = 50, learning_rate = 1.0, algorithm = ‘SAMME.R’, random_state = None
DT	criterion = ‘gini’, splitter = ‘best’, max_depth = None, min_samples_split = 2, min_samples_leaf = 1, min_weight_fraction_leaf = 0.0, max_features = None, random_state = None, max_leaf_nodes = None, min_impurity_decrease = 0.0, min_impurity_split = None, class_weight = None, ccp_alpha = 0.0
GP	kernel = None, *, optimizer = ‘fmin_l_bfgs_b’, n_restarts_optimizer = 0, max_iter_predict = 100, warm_start = False, copy_X_train = True, random_state = None, multi_class = ‘one_vs_rest’, n_jobs = None
NB	alpha = 1.0, binarize = 0.0, fit_prior = True, class_prior = None

### Evaluations

**Fig. 2:**
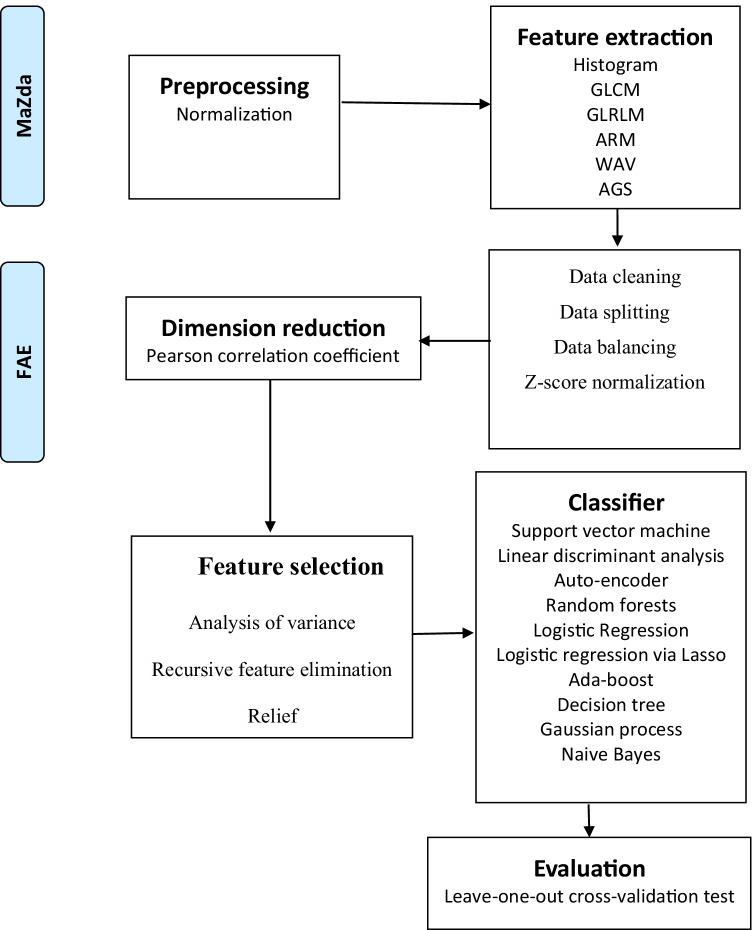
A schematic diagram for the whole radiomics and machine learning pipeline

The results were evaluated using a leave-one-out cross-validation (LOOCV) test. Using LOOCV, learning sets were created by taking all the samples except one that was used as the validation set. The accuracy, sensitivity, specificity, positive predictive value (PPV), and negative predictive value (NPV) were also calculated at a cutoff value that maximized the value of the Youden index. The area under the receiver operator characteristics curve (AUC) of the classification results was calculated for each tested condition (Fig. 2).

## Results

Of the 168 consecutive patients with a pathologic diagnosis of SRCMT and non-SRCMT over a 2-year period between January 2018 and November 2020, 16 were excluded with poor-quality images on MRI (7 were excluded due to the visible artifacts from dental work, 2 due to motion artifacts, and 7 due to visible artifacts from the bone-air interface). A total of 152 patients were finally selected for the study. There were 66 patients with SRCMT and 86 patients with non-SRCMT, specifically RMS (*n* = 14), lymphoma (*n* = 20), MM (*n* = 10), NEC (*n* = 14), ONB (*n* = 8), SCC (*n* = 62), and ACC (*n* = 24). There were 88 males and 64 females in the whole cohort. The mean age of all the patients was 54.28 years, ranging from 13 to 87 years. Seventy percent of the datasets were in the training set (106 patients; 46 with SRCMT and 60 with non-SRCMT) and 30% in the independent test set (46 patients; 20 with SRCMT and 26 with non-SRCMT).

**Fig. 3:**
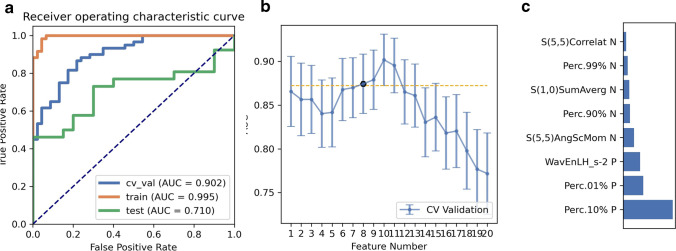
Model performance generated using recursive feature elimination. **a** Receiver operating characteristic (ROC) curves of this model using different datasets. **b** FeAture Explorer (FAE) software suggested a candidate eight-feature model according to the “one-standard error” rule. **c** The contribution of features in the final model

SMOTE was used to automatically generate 14 synthetic SRCMT samples in order to overcome the influence of imbalanced dataset on the classifier fitting. We compared the AUC of all the pipelines on the validation dataset with FAE. The pipeline using RFE feature selection and a GP classifier yielded the highest AUCs using ten features. When the “one-standard error” rule was used, FAE also produced a simpler model with eight features [21]. The ROC curves are shown in Fig. 3. The AUCs of the training, validation, and test datasets achieved 0.995, 0.902, and 0.710, respectively. FAE-selected features were Perc.01%, Perc.10%, Perc.90%, and Perc.99% from the histogram; S(1,0) SumAverg, S(5,5) AngScMom, and S(5,5) Correlat from gray-level GLCM; and WavEnLH_s-2 from wavelets transform (WAV).

**Fig. 4:**
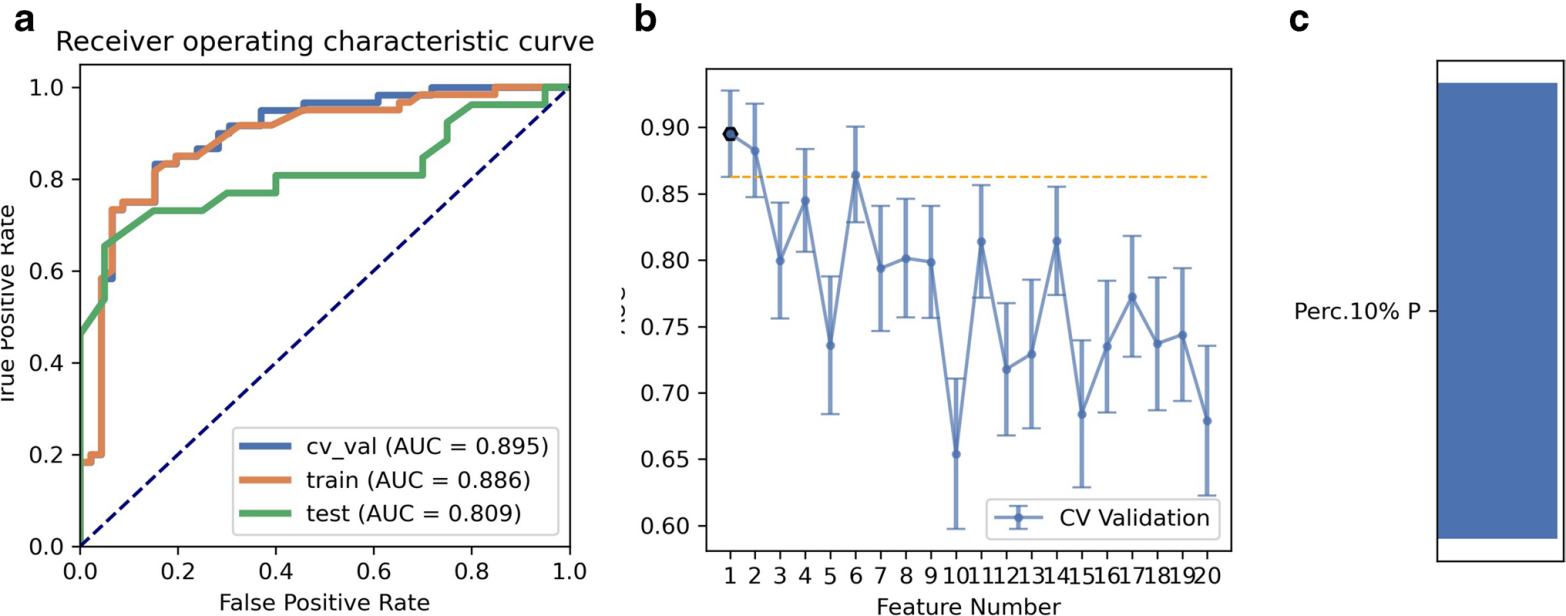
Model performance generated using the analysis of variance (ANOVA). **a** Receiver operating characteristic (ROC) curves of this model using different datasets. **b** FeAture Explorer (FAE) software suggested a candidate one-feature model according to the “one-standard error” rule. **c** The contribution of features in the final model

As for ANOVA, the pipeline using the AE classifier yielded the highest AUC using one feature with a “one-standard error” rule, as shown in Fig. 4. The AUCs of the training, validation, and test datasets achieved 0.886, 0.895, and 0.809, respectively. The FAE-selected feature was Perc.10% from the histogram (*F* = 84.24, *p* < 0.001).

**Fig. 5:**
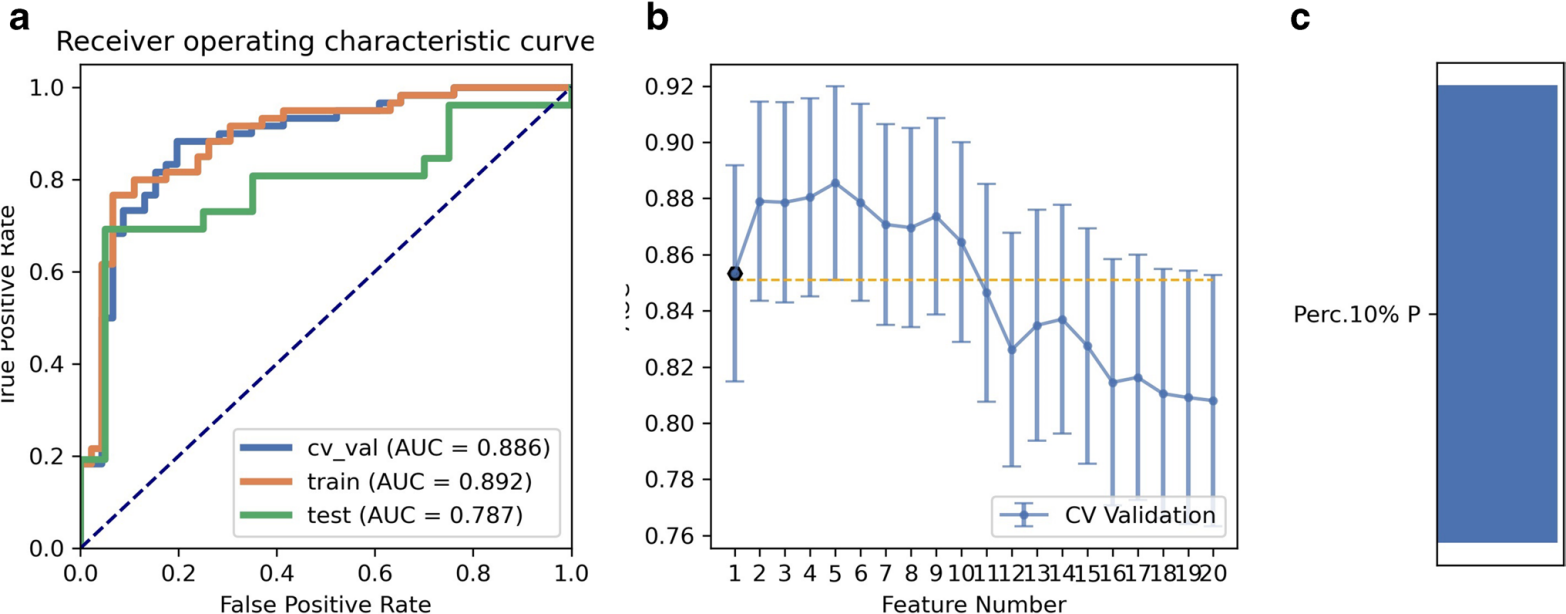
Performance of model generated by relief. **a** Receiver operating characteristic (ROC) curves of this model using different datasets. **b** FeAture Explorer (FAE) software suggested a candidate one-feature model according to the “one-standard error” rule. **c** The contribution of features in the final model

As for relief, the pipeline using the LRLasso classifier yielded the highest AUC using five features. Features selected by FAE were Perc.10% and Perc.01% from histogram, and S(4,4) Correlat, S(5,0) SumAverg, and S(5,0) Contrast from GLCM; weights were 1.09, 1.07, 0.86, 0.82, and 0.76, respectively. When the “one-standard error” rule was used, FAE also produced a simpler model with only one feature; the ROC curves are shown in Fig. 5. The AUCs of the training, validation, and test datasets achieved 0.892, 0.886, and 0.787, respectively. The feature selected from the histogram with FAE was Perc.10%.

**Fig. 6:**
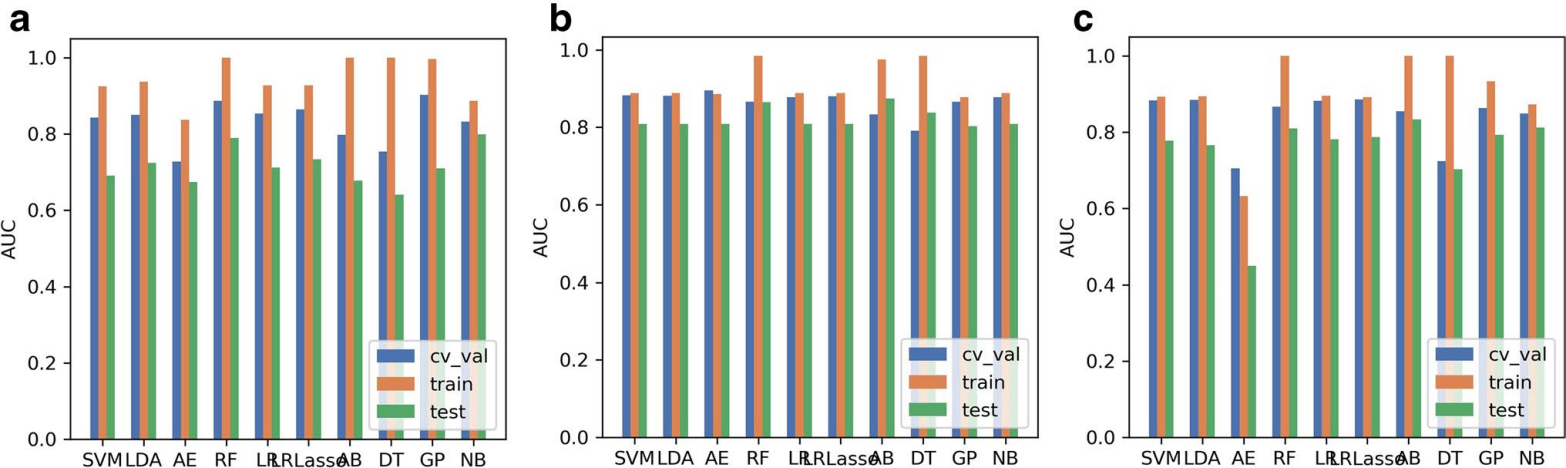
Areas under the curve (AUCs) looking at different datasets. Feature selections using (**a) **recursive feature elimination (RFE), (**b)** analysis of variance (ANOVA), and (**c)** relief

Using the RFE feature selection, the AUCs of the training, validation, and test datasets of the 10 ML algorithms were greater than 0.640 (Fig. 6A). The optimal algorithm in the validation datasets was GP, whose AUC was 0.902. In all the algorithms, the AUCs of training datasets were better than those of validation datasets whose AUCs were greater than those of the test datasets.

Using ANOVA feature selection, the AUCs of the training, validation, and test datasets of ten machine learning algorithms were greater than ~ 0.800 (Fig. 6B). The optimal algorithm in the validation datasets was AE, whose AUC was 0.895. Compared with RFE and relief, the results of all the algorithms of ANOVA feature selection were more stable.

Using relief feature selection, the AUCs of the training, validation, and test datasets of nine ML algorithms were greater than ~ 0.700 except AE (Fig. 6C). The optimal algorithm in the validation datasets was LRLasso, whose AUC was 0.886. In the nine algorithms except AE, the AUCs of training datasets were better than those of validation datasets, whose AUCs were greater than those of the test datasets.

In addition to the three feature selection methods, we also compared and listed the optimal AUCs of different ML classifications in the validation dataset (Table 3).

**Table 3:** The optimal area under the receiver operator characteristics curve (AUC), 95% confidence interval (CI), standard error, accuracy, Youden index, sensitivity, specificity, accuracy, positive predictive value (PPV), and negative predictive value (NPV) of all algorithm classifications with leave-one-out cross-validation

Feature set	AUC	95% CIs	Std	Acc	Youden Index	Sen	Spe	PPV	NPV
Zscore_PCC_ANOVA_1_AE	0.895	[0.8260–0.9533]	0.033	0.840	0.513	0.833	0.848	0.877	0.796
Zscore_PCC_ANOVA_3_LDA	0.891	[0.8212–0.9481]	0.032	0.840	0.367	0.850	0.826	0.864	0.809
Zscore_PCC_ANOVA_3_LRLasso	0.887	[0.8114–0.9512]	0.035	0.849	0.359	0.883	0.804	0.855	0.841
Zscore_PCC_ANOVA_8_SVM	0.885	[0.8100–0.9461]	0.034	0.830	0.586	0.767	0.913	0.920	0.750
Zscore_PCC_ANOVA_3_LR	0.884	[0.8103–0.9449]	0.035	0.840	0.388	0.883	0.783	0.841	0.837
Zscore_PCC_ANOVA_1_NB	0.878	[0.8025–0.9444]	0.036	0.840	0.403	0.833	0.848	0.877	0.796
Zscore_PCC_ANOVA_3_RF	0.878	[0.7977–0.9457]	0.038	0.859	0.520	0.867	0.848	0.881	0.830
Zscore_PCC_ANOVA_4_GP	0.869	[0.7940–0.9343]	0.036	0.821	0.482	0.817	0.826	0.860	0.776
Zscore_PCC_ANOVA_3_AB	0.865	[0.7867–0.9337]	0.038	0.802	0.529	0.683	0.957	0.954	0.698
Zscore_PCC_ANOVA_5_DT	0.811	[0.7298–0.8842]	0.039	0.811	1.000	0.817	0.804	0.845	0.771
Zscore_PCC_RFE_10_GP	0.902	[0.8379–0.9519]	0.029	0.830	0.498	0.867	0.783	0.839	0.818
Zscore_PCC_RFE_1_AE	0.895	[0.8260–0.9533]	0.033	0.840	0.513	0.833	0.848	0.877	0.796
Zscore_PCC_RFE_8_RF	0.894	[0.8251–0.9525]	0.033	0.849	0.565	0.833	0.870	0.893	0.800
Zscore_PCC_RFE_3_LDA	0.891	[0.8208–0.9478]	0.032	0.840	0.367	0.850	0.826	0.864	0.809
Zscore_PCC_RFE_3_LRLasso	0.886	[0.8107–0.9502]	0.035	0.849	0.359	0.883	0.804	0.855	0.841
Zscore_PCC_RFE_1_SVM	0.883	[0.8073–0.9460]	0.035	0.821	0.672	0.733	0.935	0.936	0.729
Zscore_PCC_RFE_3_LR	0.883	[0.8096–0.9449]	0.035	0.840	0.388	0.883	0.783	0.841	0.837
Zscore_PCC_RFE_1_NB	0.878	[0.8025–0.9444]	0.036	0.840	0.403	0.833	0.848	0.877	0.796
Zscore_PCC_RFE_3_AB	0.865	[0.7867–0.9337]	0.038	0.802	0.529	0.683	0.957	0.954	0.698
Zscore_PCC_RFE_9_DT	0.808	[0.7304–0.8795]	0.039	0.811	1.000	0.833	0.783	0.833	0.783
Zscore_PCC_Relief_5_LRLasso	0.886	[0.8108–0.9483]	0.035	0.849	0.359	0.883	0.804	0.855	0.841
Zscore_PCC_Relief_5_LDA	0.884	[0.8088–0.9435]	0.033	0.840	0.332	0.883	0.783	0.841	0.837
Zscore_PCC_Relief_5_SVM	0.883	[0.8077–0.9454]	0.035	0.830	0.511	0.817	0.848	0.875	0.780
Zscore_PCC_Relief_5_LR	0.882	[0.8055–0.9454]	0.035	0.830	0.417	0.850	0.804	0.850	0.804
Zscore_PCC_Relief_3_GP	0.880	[0.8054–0.9423]	0.035	0.840	0.552	0.800	0.891	0.906	0.774
Zscore_PCC_Relief_3_NB	0.875	[0.7950–0.9423]	0.037	0.830	0.362	0.817	0.848	0.875	0.780
Zscore_PCC_Relief_2_AE	0.871	[0.7907–0.9373]	0.037	0.821	0.441	0.817	0.826	0.860	0.776
Zscore_PCC_Relief_19_RF	0.869	[0.7947–0.9347]	0.035	0.821	0.645	0.767	0.891	0.902	0.746
Zscore_PCC_Relief_5_AB	0.855	[0.7652–0.9254]	0.040	0.821	0.513	0.767	0.891	0.902	0.746
Zscore_PCC_Relief_9_DT	0.786	[0.7069–0.8678]	0.041	0.793	1.000	0.833	0.739	0.807	0.773

*SVM*, support vector machine; *LDA*, linear discriminant analysis; *AE*, auto-encoder; *RF*, random forests; *LR*, logistic regression; *LRLasso*, logistic regression via Lasso; *AB*, ada-boost; *DT*, decision tree; *GP*, Gaussian process; *NB*, naive Bayes

## Discussion

This study investigated the potential value of the ADC texture analysis for distinguishing SRCMTs from non-SRCMTs by using various state-of-the-art ML algorithms. The key findings are as follows: (1) the pipeline using RFE feature selection and Gaussian process classifier yielded the highest AUC. (2) The parameter with the best diagnostic performance in differentiating SRCMTs from non-SRCMTs was the Perc.10% ADC value. (3) Results of all the algorithms of ANOVA feature selection were more stable and the AUCs were higher than 0.800, as compared with RFE and relief. (4) TA and ML appear to be the most useful in differentiating standard ADC images of maximum solid tumor components routinely acquired with high accuracy of 0.793 to 0.859.

Previous studies have shown that ADC value of malignant sinonasal lesions was significantly lower than that of benign lesions [10, 22–25] as major parts of malignant tumors were composed of high cellularity whereas ADC is inversely correlated with tissue cellularity. Consistent with this notion, Sumi et al. reported lymphomas had smaller ADCs than did well-differentiated SCC nodes in the neck [26]. Maeda et al. found a statistically significant difference in ADC values between SCCs and lymphomas in the head and neck as lymphoma cells have relatively high nuclear-to-cytoplasm ratios and are densely packed [27]. Some other studies reported ADC values of poorly differentiated and undifferentiated carcinomas were significantly lower than those of moderately differentiated and well-differentiated carcinomas of the pharynx and nasal and paranasal sinus [9, 28]. In addition, ADC levels of SRCMTs were lower than of non-SRCMTs, reflecting their cellular characteristics (undifferentiated cells with high cell attenuation, relatively small-sized nuclei, and scant cytoplasm) [8, 29]. In this study, the common parameter used to differentiate SRCMTs from non-SRCMTs was Perc.10% in the three feature selection methods. GLCM and wavelets were the other two parameters using RFE feature selection to differentiate them. These parameters were first-order, second-order, and higher order statistics which were described as the distribution of individual voxel values, statistical interrelationships between voxels with similar or dissimilar contrast values, and texture frequency component data extracted from the energy computed within channels, respectively. This indicates that histogram-based ADC parameters are more sensitive to histopathological features in sinonasal malignant tumors. We suspect that the cause of the efficacy of Perc.10% ADC lies in that it reflects the complex intratumoral microstructures and heterogeneity in the whole tumor, taking a hypoxic lesion around the tiny necrotic tissue, for example.

There would always be some features which are less important on sample sets. The least important features are pruned from current set of features. RFE feature selection addresses the problem by automatically removing these features. That procedure is recursively repeated on the pruned set until the desired number of features to select is eventually reached. GP classification is a nonparametric method based on the Laplace approximation and is used for approximating the non-Gaussian posterior using the Gaussian method. It can easily handle various problems, such as an insufficient capacity for the classical linear method, complex data types, and the curse of dimensions [30]. In this study, we used an RFE feature selection and GP classifier in a LOOCV loop to boost their performance on very high-dimensional datasets, achieving a 0.830 accuracy, 0.867 sensitivity, 0.783 specificity, and 0.902 AUC.

Our study has limitations. First, as the SRCMTs studied were of various histologic types, subgroup analyses in more details should be performed in future studies after obtaining a larger sample size and a careful consideration of the study groups. Second, as the texture-analyzing software analyzed only two-dimensional images, three-dimensional analyses which can better reflect the texture features of the entire tumor would be one of the directions of our future research. Finally, as only ADC maps were chosen, in our further studies, we will propose a multiparametric MRI investigation including ADC, T2-weighted MRI, and dynamic contrast-enhanced MRI involving early and delayed phases to generate a robust model to differentially diagnose SRCMTs and non-SRCMTs by segmenting precisely three-dimensional tumor regions in a larger sample.

## Conclusions

In this study, we investigated the feasibility of combining artificial intelligence and radiomics features from ADC values to differentially diagnose SRCMTs and non-SRCMTs. As it is non-invasive, this approach has a promising potential for future applications in clinical medicine.

## Abbreviations

AB: Ada-boost
AE: Auto-encoder
AGS: Absolute gradient statistics
ANOVA: Analysis of variance
ARM: Auto-regressive model
DT: Decision tree
GLCM: Gray-level co-occurrence matrix
GLRLM: Gray-level run-length matrix
GP: Gaussian process
LDA: Linear discriminant analysis
LR: Logistic regression
LRLasso: Logistic regression via Lasso
ML: Machine learning
NB: Naive Bayes
PCC: Pearson correlation coefficient
RF: Random forests
RFE: Recursive feature elimination
SMOTE: Synthetic minority oversampling technique
SRCMTs: Small round cell malignant tumors
SVM: Support vector machine
TA: Texture analysis
WAV: Wavelet transform

## Declarations

### Guarantor

The scientific guarantor of this publication is Prof. Fabao Gao.

### Statistics and biometry

Fabao Gao, PhD, kindly provided statistical advice for this manuscript.

### Methodology

• retrospective

• diagnostic study

• performed at one institution
